# Supplementary material for: Suppressing mosquito populations with precision guided sterile males
Source: Nat Commun. 2021 Sep 10;12:5374. doi: 10.1038/s41467-021-25421-w (PMC8433431; doi:10.1038/s41467-021-25421-w)
Supplement: Supplementary file 1 — Supplementary Information [file 41467_2021_25421_MOESM1_ESM.pdf]

Supplementary Figures

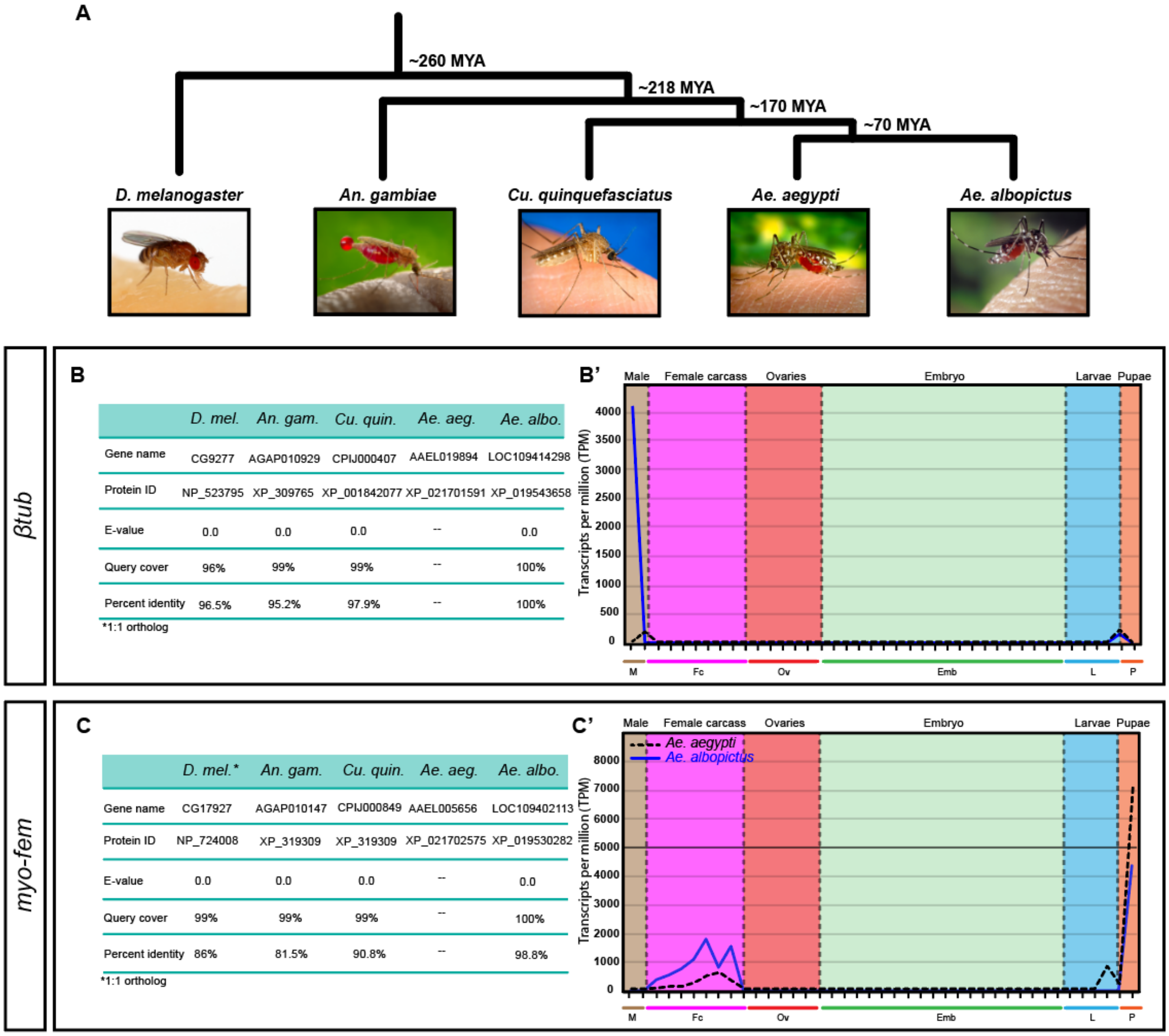

Supplementary Figure 1. Conservation of target genes in Diptera. (A) Phylogenetic tree of Dipteran insects, including *Drosophila melanogaster (D. melanogaster and D. mel.)*, *Anopheles gambiae (An. gambiae* and *An. gam.)*, *Culex quinquefasciatus (Cu. quinquefasciatus* and *Cu. quin.)*, *Aedes aegypti (Ae. aegypti* and *Ae. aeg)*, and *Aedes albopictus (Ae. albopictus* and *Ae. albo)*, with evolutionary distance measured by million years ago (MYA). All images are free to use and were downloaded from wiki commons, except *Cu. quin.,* which was downloaded from Pixnio.com. (B) *βTub* orthologs in Dipteran species with highest similarity to the *Ae. aegypti βTub* protein at the amino acid level. (B') RNA expression levels of the *βTub* gene throughout development. Extremely high expression of *βTub* is seen in *Ae. albopictus* testes samples (refer to Table S1 for TPM data of *βTub* expression across development). Lower gene expression is observed in both ♂ carcass and ♂ pupae samples. (C) *myo-fem* orthologs in Dipteran species with the highest similarity to the *Ae. aegypti myo-fem* protein at the amino acid level. (C') RNA expression levels of *myo-fem* across development in both *Ae. aegypti* and *Ae. albopictus* mosquito samples. The *myo-fem* gene is highly expressed in the pupal samples of both mosquito species (refer to Table S1 for TPM data of *myo-fem* expression across development). (B' and C') RNA-Seq expression levels of *myo-fem* and *βTub* genes in *Ae. aegypti* and *Ae. albopictus* mosquito samples using available data [13,14]. Major stages of development are color coded, where brown represents ♂ testes and ♂ carcass samples, pink represents ♀ carcass samples, red represents ovary samples, green represents

embryogenesis, blue represents larval samples, and orange represents pupae samples. Black dotted lines represent *Ae. aegypti,* and solid blue lines represent *Ae. albopictus*. The major developmental groups are indicated by color bars and are organized left to right, as follows: M (brown, ♂ testes, ♂ carcass), Fc (purple, NBF ♀ Carcass, and multiple time points PBM: 12, 24, 36, 48, 60, and 72 hr), Ov (red, NBF ovaries, and multiple ovarian time points PBM: 12, 24, 36, 48, 60, and 72 hr), Emb (green, embryo, 0–2, 2–4, 4–8, 8–12, 12–16, 16–20, 20–24, 24–28, 28–32, 32–36, 36–40, 40–44, 44–48, 48–52, 52–56, 56–60, 60–64, 64–68, 68–72, and 72–76 hr embryos), L (light blue, larvae, 1st, 2nd, 3rd, and 4th instar larvae stages), and P (light orange, ♂ and ♀ pupae). *1:1 orthologs. Data can be found in Supplemental Table 1. Source data are provided as a Source Data file.

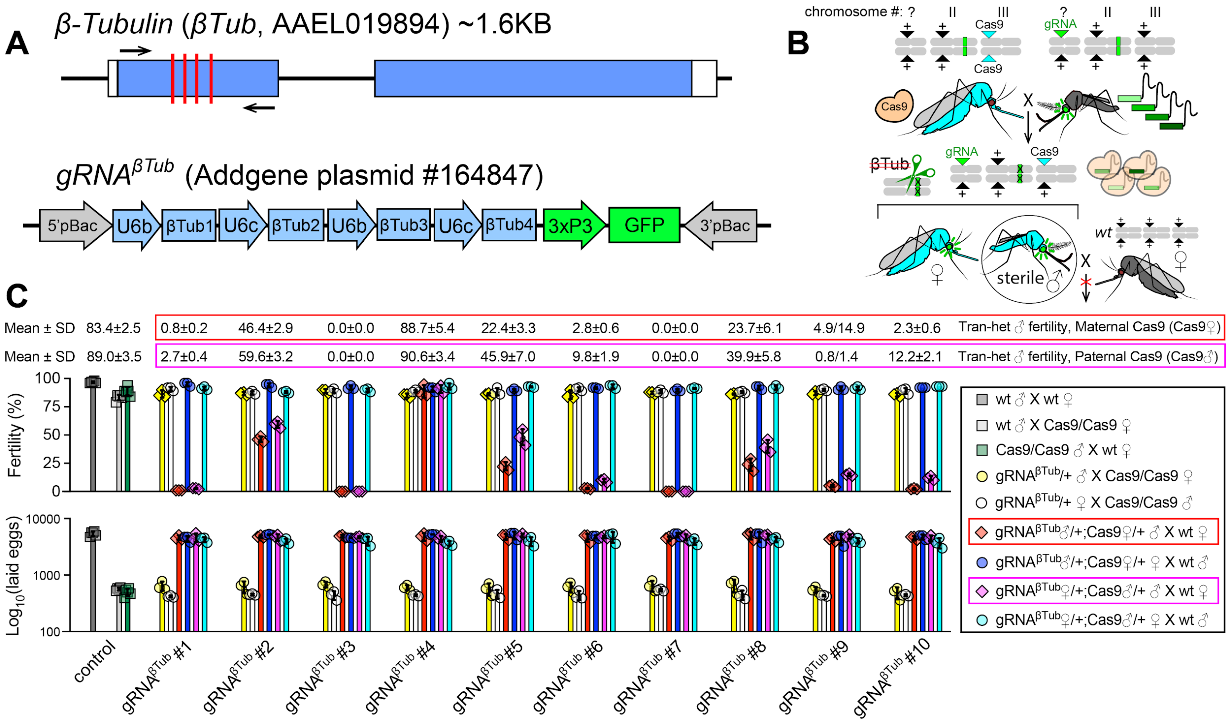

Supplementary Figure 2. Assessment of independent *gRNA$^{\beta Tub}$* lines. (A) Schematic of the *Ae. aegypti β-Tubulin* (*βTub*) locus and the *gRNA$^{\beta Tub}$* construct used to generate 10 transgenic *gRNA$^{\beta Tub}$* lines. The *gRNA$^{\beta Tub}$* construct harbors four independent gRNAs targeting different sequences in the 1st coding exon of *βTub* (red lines) and a *3xP3-GFP* marker gene. (B) A schematic of the genetic cross between the homozygous *Cas9* ♀'s and heterozygous *gRNA$^{\beta Tub}$/+* ♂'s generating the transheterozygous progeny inheriting maternal Cas9. To assess the efficiency of *βTub* disruption in F_1 transheterozygous progeny, this cross was set up reciprocally between the homozygous *Cas9* line and each of ten different insertion lines of *gRNA$^{\beta Tub}$*. The generated F_1 transheterozygous ♂'s and ♀'s were crossed to WT mosquitoes of the opposite sex, and their fertility and fecundity, as an average number of laid eggs, were compared to those of F_0 parents, homozygous *Cas9*, and WT mosquitoes. (C) The bar plots show at least three ($n \geq 3$) biological replicates and means ± SDs for fertility and fecundity (Log_{10}[laid eggs]) of tested groups (Table S3). Different insertion lines of the same *gRNA$^{\beta Tub}$* construct in conjunction with *Cas9* induced a range of fertility in transheterozygous ♂'s from 90.6 ± 3.4% to 0%. Two lines, *gRNA$^{\beta Tub}$#3* and *gRNA$^{\beta Tub}$#7*, independently induced the robust sterility of transheterozygous ♂'s where they harbored maternal or paternal Cas9: *gRNA$^{\beta Tub}$♂/+; Cas9♀/+* or *gRNA$^{\beta Tub}$♀/+; Cas9♂/+*, respectively. The *gRNA$^{\beta Tub}$#7* was the easiest to score due to its brighter expression of the *3xP3-GFP* marker. Therefore, it was used for further analysis and genetically combined with the best *gRNA$^{myo\text{-}fem}$* line. Data can be found in Supplemental Table 3. Source data are provided as a Source Data file.

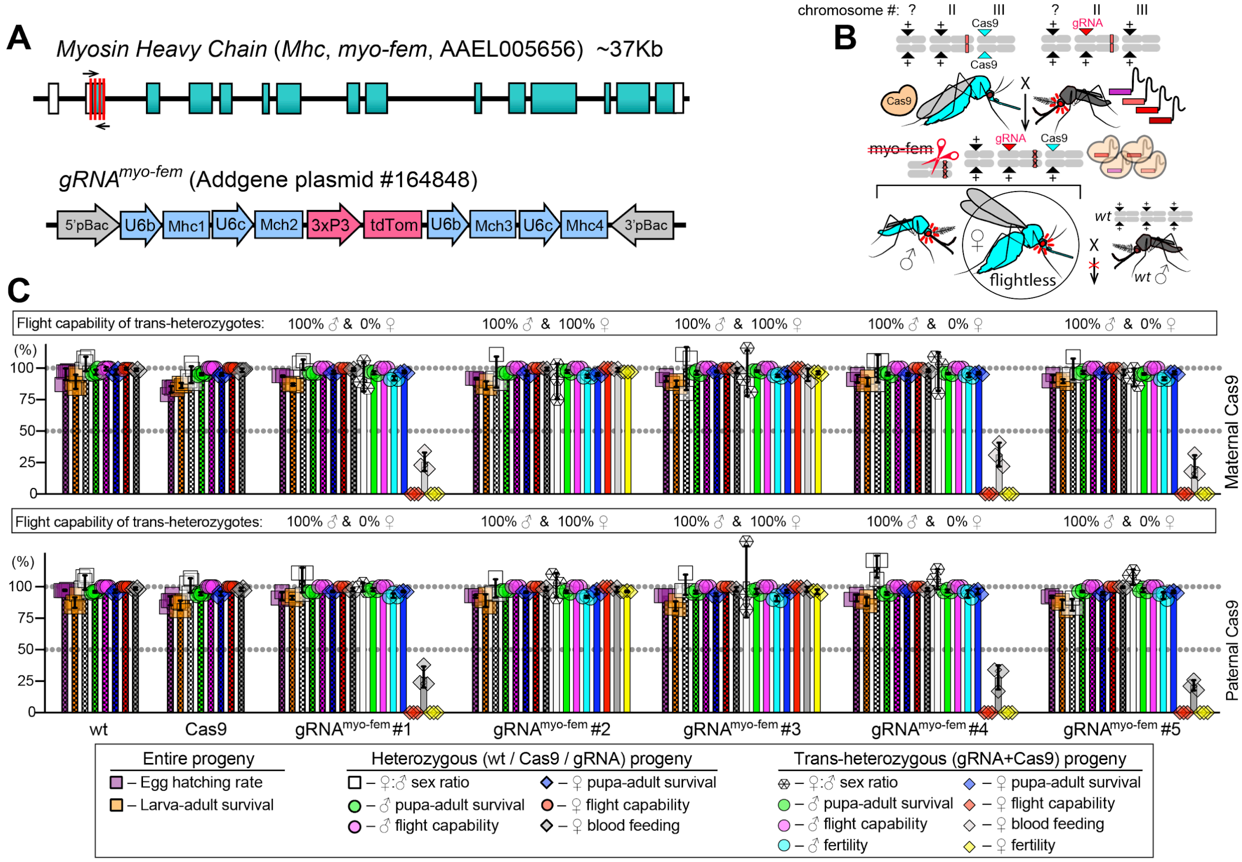

Supplementary Figure 3. Assessment of independent *gRNA$^{myo\text{-}fem}$* lines. (A) Schematic of the *Ae. aegypti Myosin Heavy chain* (*Mhc, myo-fem*) locus and the *gRNA$^{myo\text{-}fem}$* genetic construct used to generate five transgenic *gRNA$^{myo\text{-}fem}$* lines. The *gRNA$^{myo\text{-}fem}$* construct harbors four independent gRNAs targeting different sequences in the 1st exon of *myo-fem* (red lines) and a *3xP3-tdTomato* marker gene. (B) A schematic of the genetic cross between the homozygous *Cas9* ♀'s and heterozygous *gRNA$^{myo\text{-}fem}$/+* ♂'s generating the transheterozygous progeny inheriting maternal Cas9. To assess the efficiency of *myo-fem* disruption in F_1 transheterozygous progeny, this cross was set up in both reciprocal directions between the homozygous *Cas9* line and each of five different insertion lines of *gRNA$^{myo\text{-}fem}$*. We scored the flight ability, pupal lethality, blood feeding, and fertility in generated F_1 transheterozygous ♂'s and ♀'s and compared to WT and Cas9 mosquitoes. (C) The bar plots show at least three ($n \geq 3$) biological replicates and means ± SDs for assessed characteristics (Table S3). In the presence of *Cas9*, three out of five *gRNA$^{\beta Tub}$* lines induced the *myo-fem* disruption that resulted in the complete ♀-specific flightlessness, while transheterozygous ♂'s were able to fly. The ♀ inability to fly affected their blood feeding, mating, and survival, rendering transheterozygous ♀'s infertile. The *gRNA$^{myo\text{-}fem}$#1* was the easiest to score due to its brighter expression of the *3xP3-tdTomato* marker. Therefore, it was used for further analysis and genetically combined with the *gRNA$^{\beta Tub}$#7* line. Data can be found in Supplemental Table 3. Source data are provided as a Source Data file.

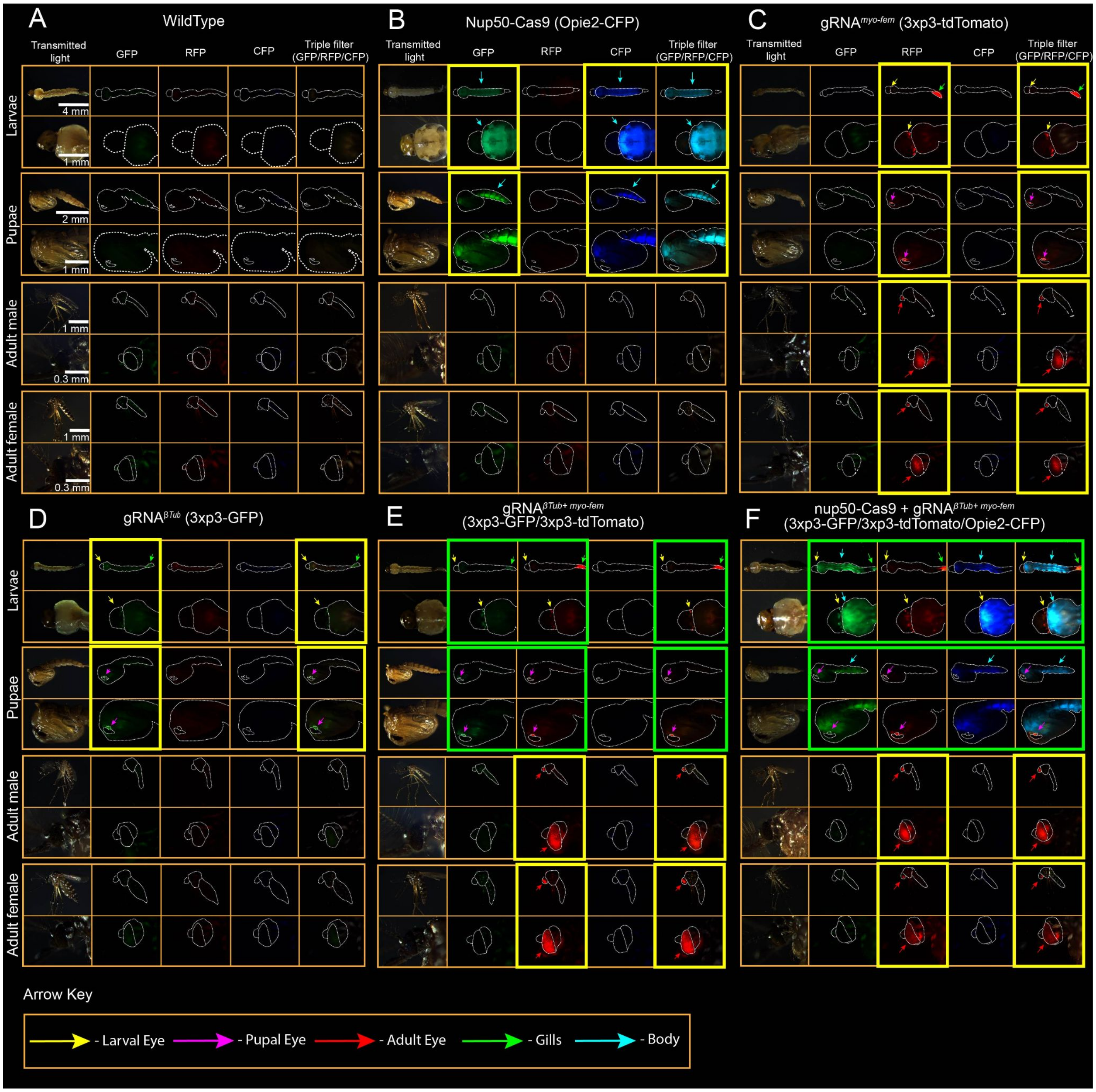

Supplementary Figure 4. Transmitted light and fluorescent images of mosquito life stages of strains used in this study. For each strain, multiple life stages are shown, including larvae (instar 4), pupae, and adult ♂'s and ♀'s. All stages are imaged using transmitted white light, GFP filter (Leica Part #10447408; ET470/40x, ET525/50m wavelengths), RFP filter (Leica Part #10450195; ET560/40x, ET630/75m wavelengths), CFP filter (Leica Part #10447409; ET436/20x, ET480/40m wavelengths) and a triple filter for GFP/RFP/CFP (Leica Part #10450611; ET434.5/21, 501.5/19, 574.5/23, ET469.5/25, 536.5/29, 635.5/69 wavelengths) using a Leica M165FC fluorescent stereomicroscope. (A) WT mosquitoes. (B) *Cas9* marked with *Opie2-CFP*. (C) *gRNA$^{myo-fem}$* marked with *3xp3-tdTomato*. (D) *gRNA$^{\beta Tub}$* marked with *3xp3-GFP*. (E) *gRNA$^{\beta Tub+myo-fem}$* marked with *3xp3-GFP* and *3xp3-tdTomato*. (F) *Cas9* + g*RNA$^{\beta Tub+myo-fem}$* marked with *Opie2-CFP*, *3xp3-GFP*, and *3xp3-tdTomato*. Arrows point to where fluorescent markers can easily be distinguished, including the eye, gills and body. Yellow box indicates one fluorescent marker can be easily distinguished under a fluorescent filter. Green box indicates multiple fluorescent markers can be easily distinguished under a fluorescent filter. Fluorescent markers and their corresponding expression patterns were consistently observed throughout all stages of screening and backcrossing of generated transgenic lines (n>3000).

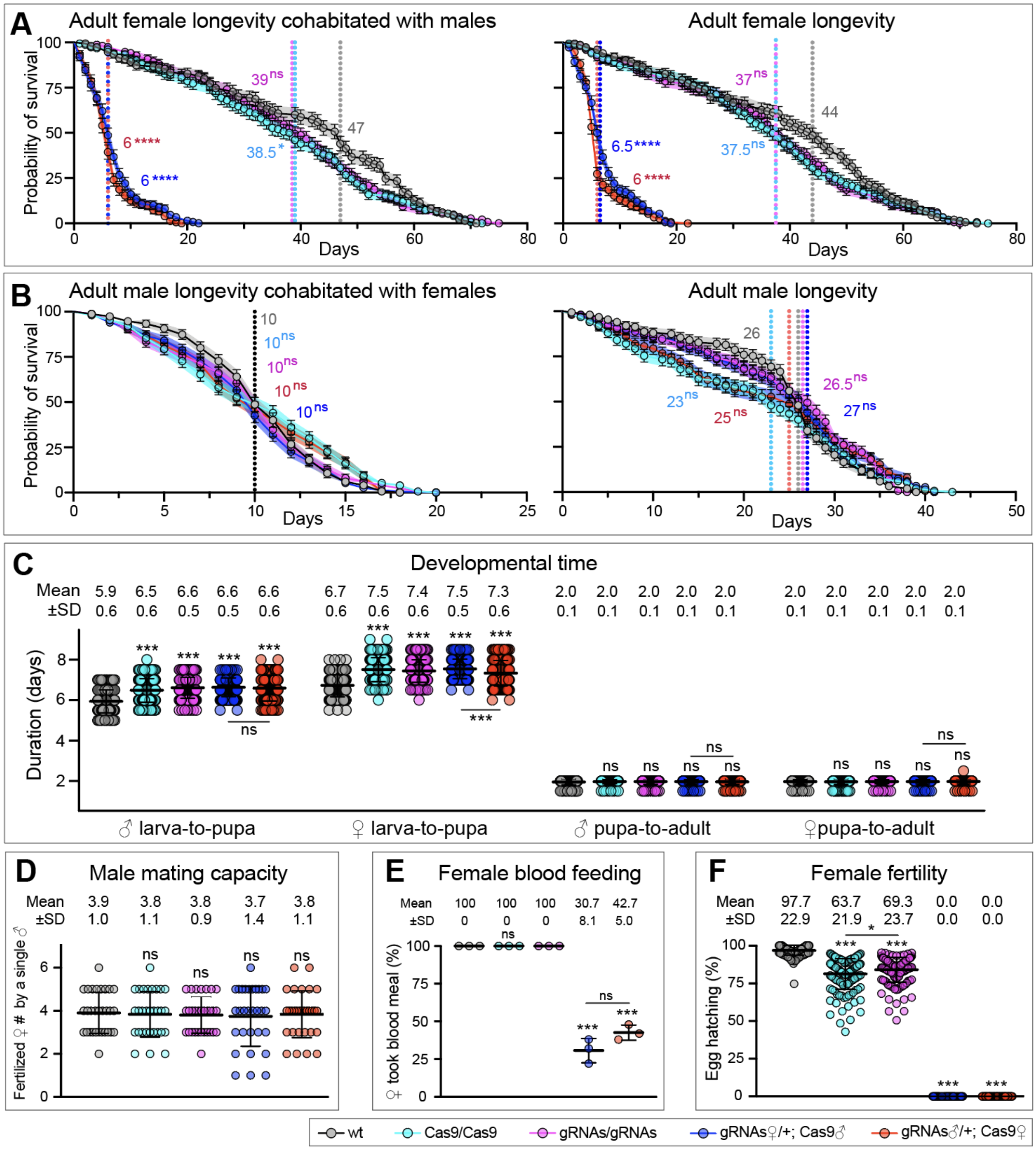

Supplementary Figure 5. Fitness of transheterozygous pgSIT mosquitoes in comparison with WT and parental lines. Survival plots of 50 adult ♀'s either cohabitated with ♂'s or not (A), and 50 adult ♂'s either cohabitated with ♀'s or not (B) over three independent experiments (n=3). Survival means ± standard errors (SE) over days following adult eclosion are plotted. Vertical lines and values present median survivals for each tested group. Survival curves were compared to the curve for WT's of the corresponding sex. The departure significance was assessed with the Log-rank (Mantel-Cox) test and is indicated above median values. The flightlessness of *pgSIT*♀'s affected their survival and drastically reduced their longevity even in the laboratory setting. Notably, the longevity of *pgSIT*♂'s was not significantly affected. (C) Plots of larva-to-pupa and pupa-to-adult developmental times were measured in 150 ♀'s and ♂'s (n=150). (D) Plots of mating capacities for 30 adult ♂'s of each genotype (n=30). (E) Plots of blood feeding

rates in 3 groups of 50 adult ♀'s of each genotype (n=3). (F) Female fertility plots, measured as egg hatching rates, of 150 adult ♀'s of each genotype (n=150). Point plots in panels C, D, E, and F show mean ± standard deviation (SD) (Table S5). A two-sided F test was used to assess the variance equality. Statistical significance of mean differences was estimated using a two-sided Student's t test with unequal or equal variance.($p \geq 0.05^{ns}$, $p < 0.05$*, $p < 0.01$**, $p < 0.001$***, and $p < 0.0001$****). Data can be found in Supplemental Table 5. Source data are provided as a Source Data file.

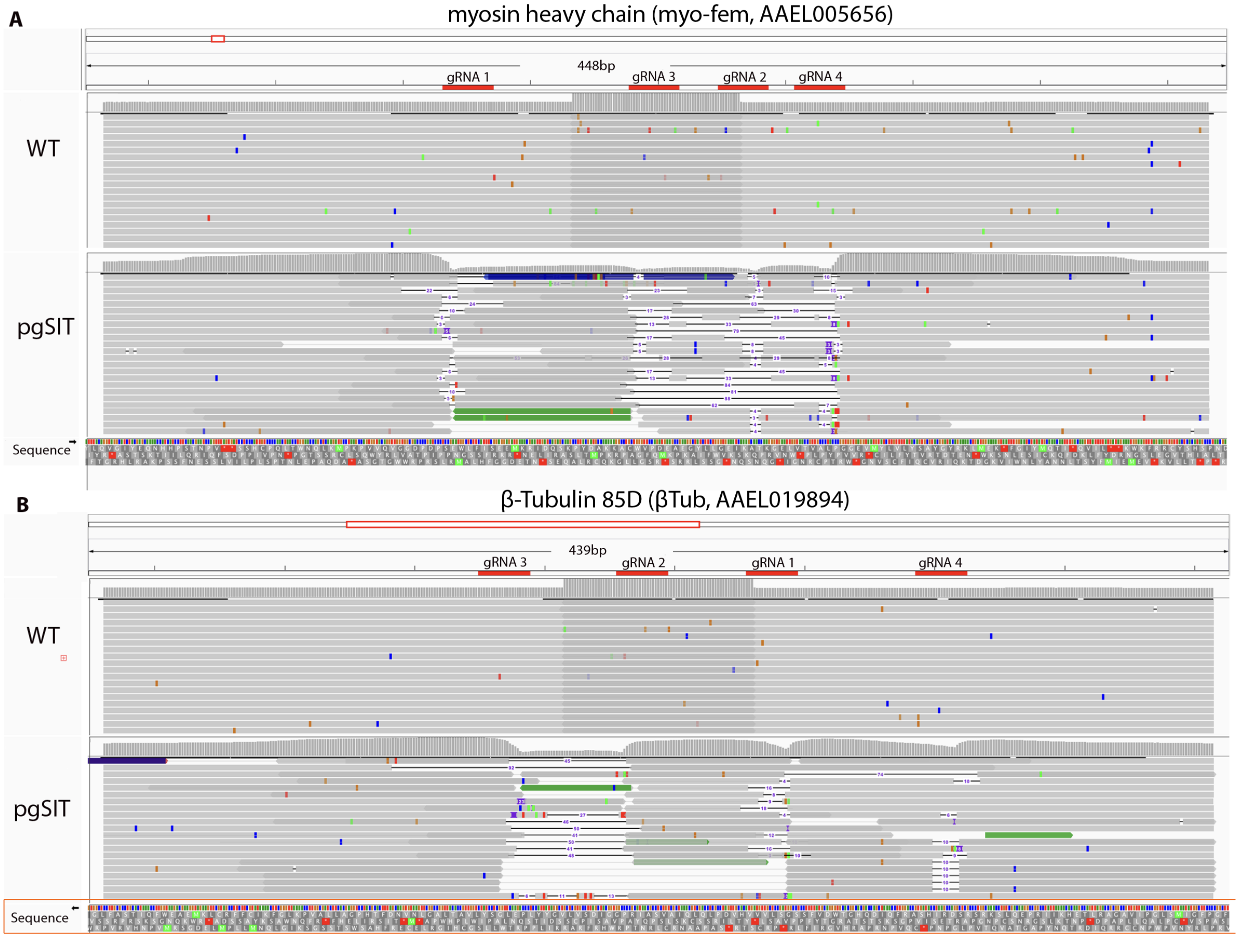

Supplementary Figure 6. Illumina NGS-based amplicon sequencing results representing *myo-fem* and *βTub* knockout in pgSIT mosquitoes. A zoomed in genome browser snapshot depicting amplicon sequencing based insertions/deletions (indels) at each gRNA target site of: (A) *myo-fem* exon 1 in WT individuals (25♀ + 25♂) and pgSIT individuals (25♀ + 25♂). (B) *βTub* exon 1 in WT individuals (25♀ + 25♂) and pgSIT individuals (25♀ + 25♂).

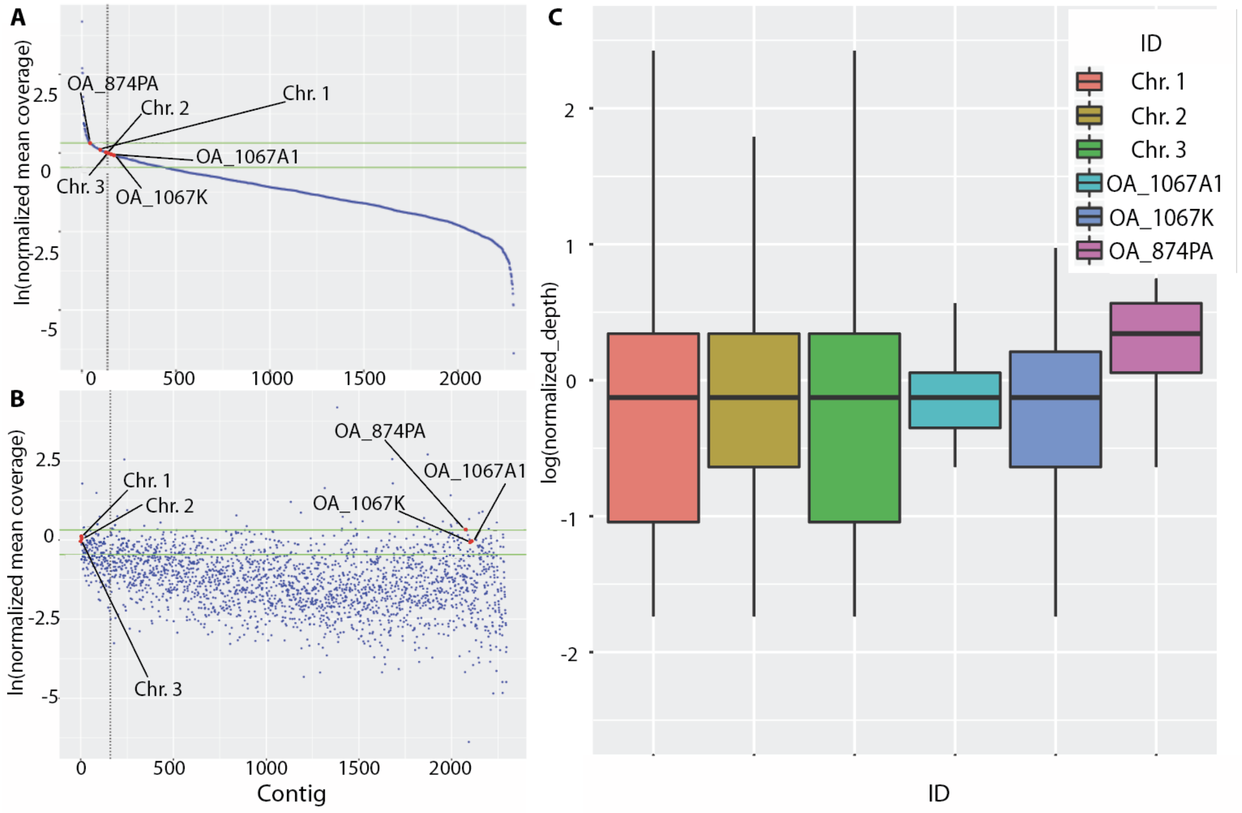

Supplementary Figure 7. Determination of transgene copy number using Oxford Nanopore genome sequencing. The normalized mean coverage for all the contigs in the genome with the exception of mtDNA, which has a coverage of 6563. These are plotted (blue) by sorting the order of the contigs as either (A) a mean coverage to produce a smooth line or (B) by contig size. The green horizontal lines correspond to the standard deviation. (C) A standard box plot depicting the coverage distributions of the three chromosomes and the three transgenes. The center line is median, first and third quartiles are the bounds of the box, upper and lower whiskers extend from the box to the largest and lowest observed value, but no further than 1.5* Inter-Quartile Range (IQR) from the box. Data associated with this figure can be found in Supplemental Tables 9-10. Source data are provided as a Source Data file.

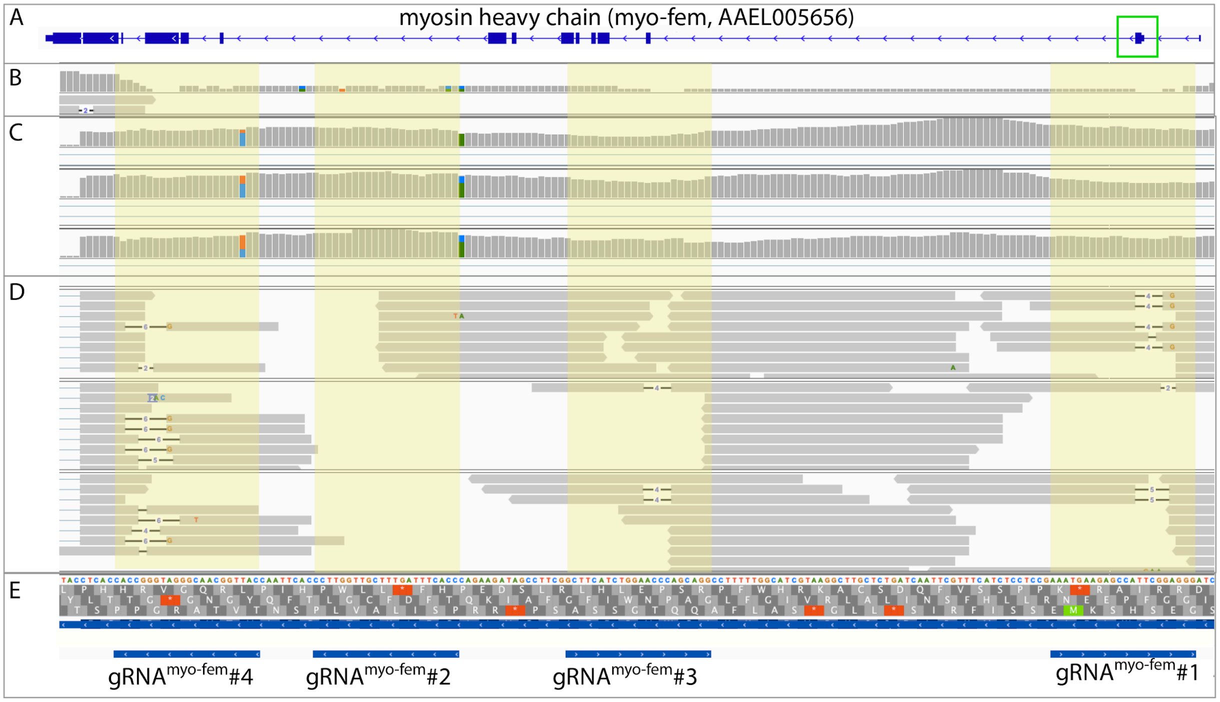

Supplementary Figure 8. Integrated genome browser snapshot depicting pgSIT sequencing results for *myo-fem*. (A) A zoomed out image of the *myo-fem* gene structure with a green box highlighting exon 1 targeted by the gRNAs. (B-E) A zoomed in genome browser snapshot of exon 1 depicting: (B) Oxford nanopore sequencing results depicting CRISPR/Cas9-mediated mutations in the DNA sequence of *myo-fem* exon 1. (C) Illumina transcriptome RNA-sequencing results of WT sequences (3 replicates) showing the lack of mutations as compared to Illumina transcriptome RNA-sequencing results in (D) depicting mutations in the coding sequence of *myo-fem* exon 1 in pgSIT individuals (3 replicates). (E) Depicts the precise locations of the four gRNA target sites.

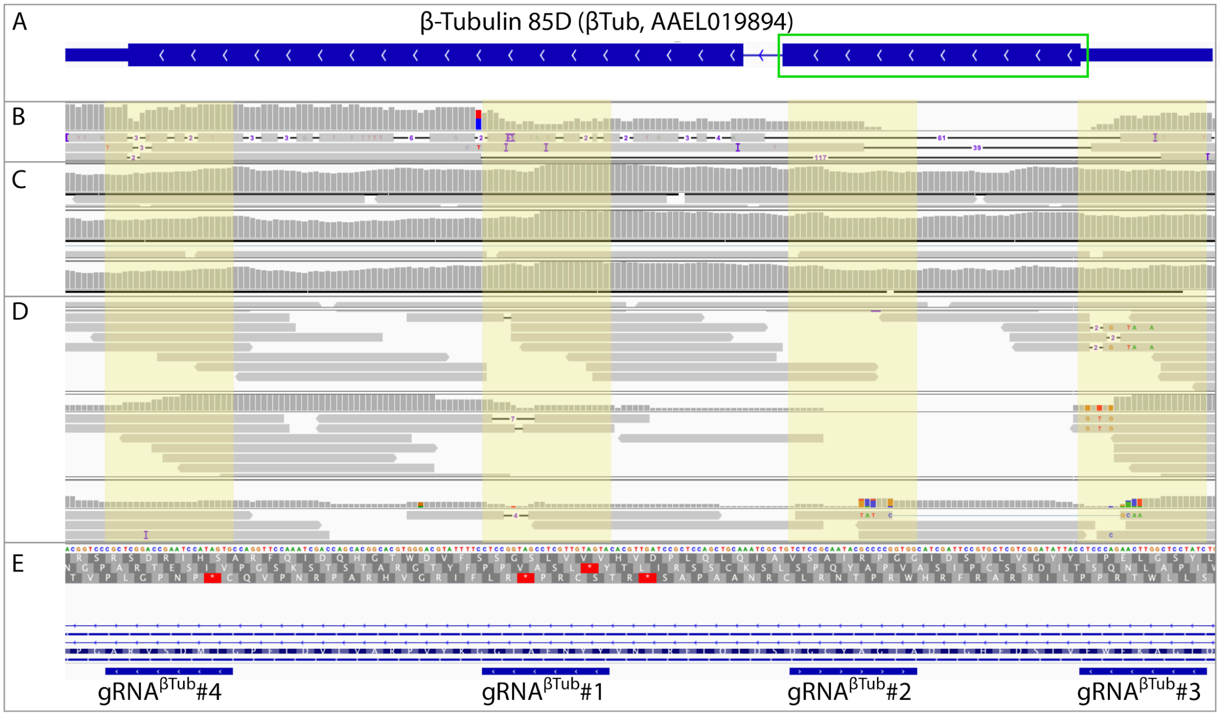

Supplementary Figure 9. Integrated genome browser snapshot depicting pgSIT sequencing results for *βTub*. A genome browser snapshot of *βTub* depicting: (A) A zoomed out image of the *βTub* gene structure with a green box highlighting the exon targeted by gRNAs. (B-E) A zoomed in genome browser snapshot of exon-1 depicting: (B) Oxford nanopore sequencing results depicting disruptions in the DNA sequence of *βTub* exon 1. (C) Illumina transcriptome RNA-sequencing of WT sequences (three replicates) showing the lack of mutations as compared to (D) depicting mutations in the coding sequence of *βTub* exon 1 in pgSIT individuals (three replicates). (E) Depicts the precise locations of the four gRNA target sites.

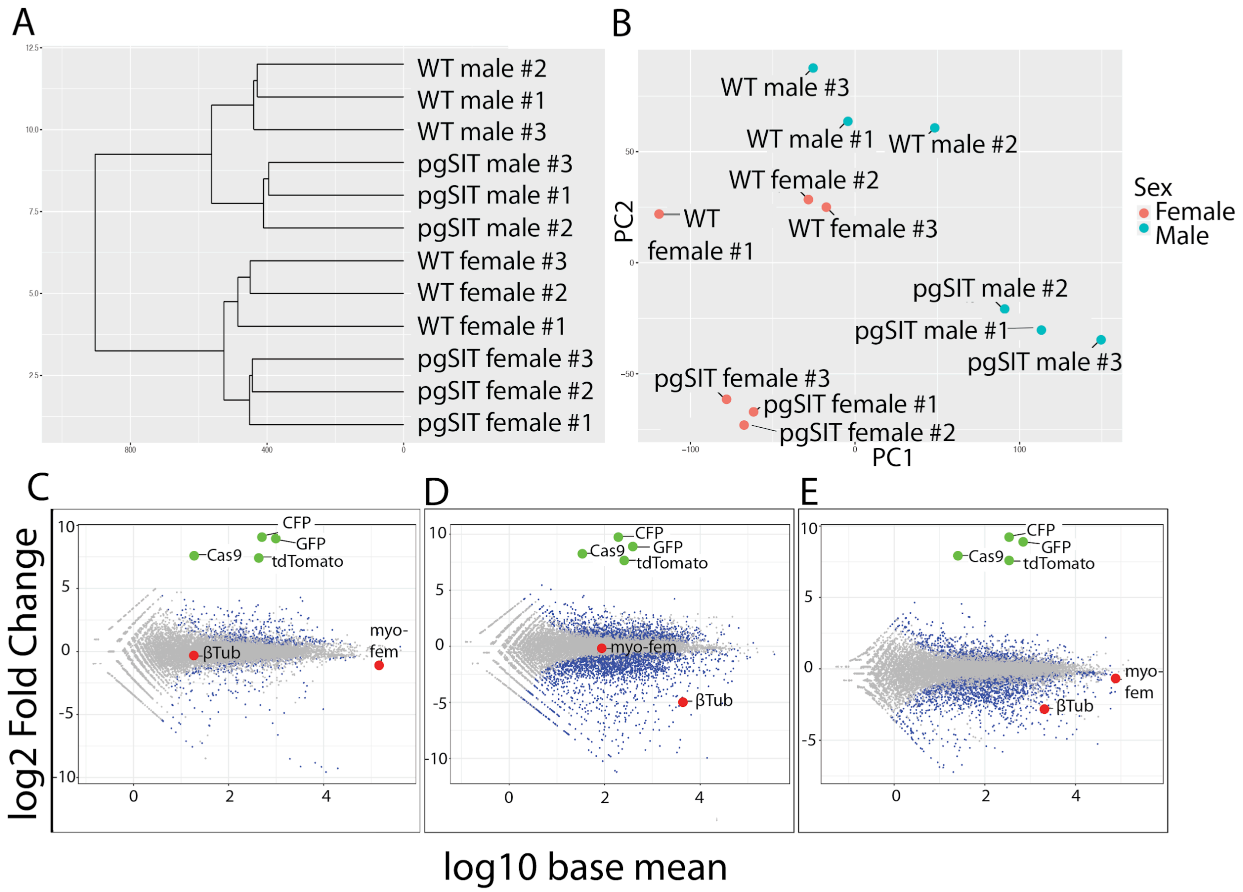

Supplementary Figure 10. Transcriptional profiling and expression analysis. (A) Hierarchical clustering and (B) PCA analysis of the 12 samples used for RNA sequencing. (C-E) MA-plots showing the differential expression patterns between: (C) *pgSIT*♀ vs WT ♀, (D) *pgSIT*♂'s vs WT ♂, (E) two-factor *pgSIT* vs WT. Significantly differentially expressed genes are indicated by blue dots (FDR < 0.5), non-significantly differentially expressed genes are indicated by grey dots, target genes are indicated by red dots, transgene encoded genes are indicated by green dots. Data associated with this figure can be found in Supplementary Tables 11-15. Source data are provided as a Source Data file.

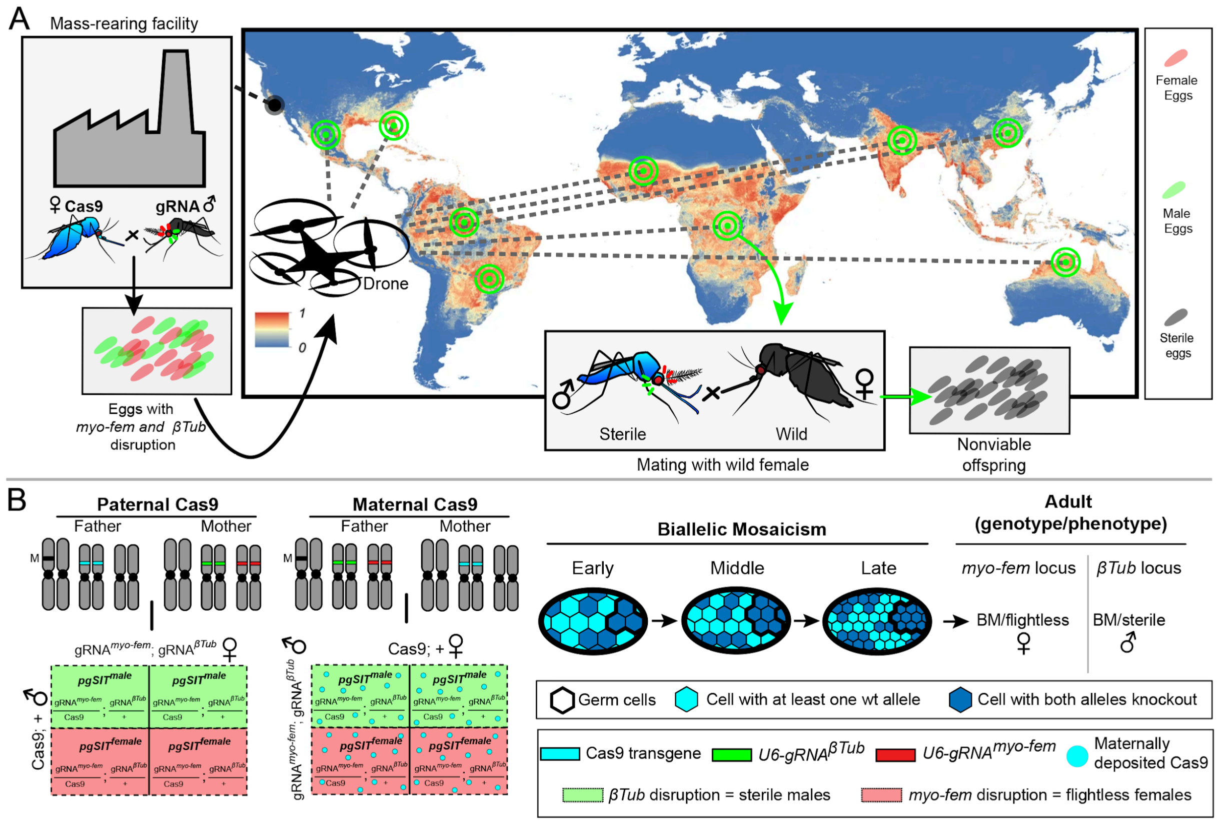

Supplementary Figure 11. Scaling pgSIT to control populations of mosquitoes and molecular mechanisms. (A) A factory produces pgSIT eggs for distribution and release at remote locations worldwide. The global map depicts the probability of occurrence of *Ae. aegypti* (from 0 blue to 1 red) at a spatial resolution of 5 km × 5 km (adopted from [42]). (B) Punnett squares depict the F1 genotypes derived from bidirectional crosses between homozygous $gRNA^{\beta Tub+myo\text{-}fem}$ and *Cas9*. To the right, is a schematic depicting the genetic outcome of a pgSIT cross and the biallelic mosaicism mechanism ensuring F1 flightless ♀ and sterile ♂ phenotypes [11].
