# Supplementary material for: Suppressing mosquito populations with precision guided sterile males
Source: Nat Commun. 2021 Sep 10;12:5374. doi: 10.1038/s41467-021-25421-w (PMC8433431; doi:10.1038/s41467-021-25421-w)
Supplement: Supplementary file 12 — List of Supplementary Files [file 41467_2021_25421_MOESM12_ESM.docx]

**Supplemental Data**

**Supplemental Table 1. *myo-fem* and *βTub* developmental gene expression data.** *Ae. albopictus* and *Ae. aegypti* *myo-fem* and *βTub* TPM gene expression across different developmental timepoints

**Supplemental Table 2. Embryo microinjection and transgenic line generation**

**Supplemental Table 3. Single gene disruption**

**Supplemental Table 4. pgSIT cross data**

**Supplemental Table 5. Life Parameters.** Comparisons of life parameters of different mosquito lines. To evaluate the potential fitness costs associated with the pgSIT components, several life-table parameters such as fecundity, larval development time, ♂ insemination capacity, mating competitiveness, and adult survival rate were measured among WT, homozygous *gRNA^βTub^*^+^*^myo-fem^*, homozygous *Cas9* and heterozygous pgSIT lines (*gRNA^βTub+myo-fem^,+/Cas9,+*). Compared to WT, homozygous *gRNA^βTub^*^+^*^myo-fem^*, homozygous *Cas9*, and *pgSIT^♂^*’s that produced from maternal Cas9 or paternal Cas9 had no significant differences in larval and pupal survival rate, larval and pupal development time, ♂ insemination ability, and adult survival (*p* < 0.05). Maternal Cas9 refers to paternal *gRNA^βTub^*/+; *gRNA^myo-fem^*/+; maternal *Cas9*/+, and paternal Cas9 corresponds to maternal *gRNA^βTub^*/+; *gRNA^myo-fem^*/+; paternal *Cas9*/+. Each strain is labeled with a superscript (a-e) and is used to indicate where the significance lies. For example, an ANOVA with a post hoc Tukey’s analysis for ♀ fecundity indicates differences are between WT vs gRNA, WT vs Cas9, and WT vs transheterozygotes.

**Supplemental Table 6. pgSIT flight capacity assay.** Flight activities were monitored over a 24-hour period using the DAM system. The counts are the number of times the mosquitoes passed the infrared beam.

**Supplemental Table 7. Sound attraction assay**

**Supplemental Table 8. pgSIT^♂^ sterilize WT ♀’s**

**Supplemental Table 9.** The mean coverage depth from the Nanopore DNA sequencing for all contigs in the genome (2310) and the three plasmids (OA-1067A1: *gRNA^βTub^*; OA-1067K: *gRNA^myo-fem^*; and OA-874PA: *Nup50-Cas9*) as well as normalized coverage based on the global number (**Table S12**). Transgene coverage ranged from 5.1 to 7.6 and normalized coverage ranged from 0.93 to 1.38.

**Supplemental Table 10. Nanopore coverage means**

**Supplemental Table 11. Mapping Stats for RNA sequencing**

**Supplemental Table 12. RNAseq expression data**

**Supplemental Table 13. deseq2_liverpool_males_pgSIT_males.annotations**

**Supplemental Table 14. deseq2_liverpool_females_pgSIT_females.annotations.xlsx**

**Supplemental Table 15. deseq2_liverpool_pgSIT.annotations**

**Supplemental Table 16. Multigenerational population cage data**

**Supplemental Table 17. Parameters used in *Aedes aegypti* population suppression model.**

**Supplemental Table 18. Primer and gRNA sequences**

**Supplemental Figures**

**Supplemental Figure 1.** Conservation of target genes in Diptera.

**Supplemental Figure 2.** Assessment of independent *gRNA^βTub^* lines.

**Supplemental Figure 3.** Assessment of independent *gRNA^myo-fem^* lines.

**Supplemental Figure 4.** Transmitted light and fluorescent images of mosquito life stages of strains used in this study.

**Supplemental Figure 5.** Fitness of transheterozygous pgSIT mosquitoes in comparison with WT and parental lines.

**Supplemental Figure 6.** Illumina NGS-based amplicon sequencing results representing *myo-fem* and *βTub* knockout in pgSIT mosquitoes.

**Supplemental Figure 7.** Determination of transgene copy number using Oxford Nanopore genome sequencing.

**Supplemental Figure 8.** Integrated genome browser snapshot depicting pgSIT sequencing results for *myo-fem*.

**Supplemental Figure 9.** Integrated genome browser snapshot depicting pgSIT sequencing results for *βTub*.

**Supplemental Figure 10.** Transcriptional profiling and expression analysis.

**Supplemental Figure 11.** Scaling pgSIT to control populations of mosquitoes and molecular mechanisms.

**Supplemental Videos**

**Supplemental Movie 1. Timelapse of *βTub* mutant and WT testes and sperm.** *βTub* mutant and WT testes were imaged at 10X and 63X.

**Supplemental Movie 2. *myo-fem* mutant ♀’s eclosing.** Flightless *myo-fem* mutant ♀’s have abnormal wing postures restricting their escape from rearing cups following eclosion, which reduces survival.

**Supplemental Movie 3. Timelapse of *myo-fem* mutant flight.** Cages consisting of *myo-fem* mutant ♀’s, *myo-fem* mutant ♂’s, WT ♀’s, and WT ♂’s were recorded over 5.5 minutes. The cages were occasionally tapped to stimulate movement.

**Supplemental Movie 4. Timelapse of pgSIT and WT mosquitoes.** Cages consisting of pgSIT mutant ♀’s, *pgSIT^♂^*’s, WT ♀’s, and WT ♂’s were recorded for 5.5 minutes. The cages were occasionally tapped to stimulate movement/flight.

**Supplemental Movie 5 DAM assay video.** Short clip of the DAM assay’s monitor tube in action with a WT ♀ passing the infrared beam during flight.

**Supplemental Movie 6.** ♂ **courtship assay**. pgSIT ♂’s are strongly attracted to the ♀ flight tone indicating strong mating behavior.

**Supplemental Movie 7.** **Model-predicted impact of releases of *pgSIT* eggs in Onetahi, Teti’aroa, French Polynesia.** Time-series for ♀ *Ae. aegypti* population density and elimination probability are depicted for four sample release schemes depicted in **Figure 4**.

**Supplemental Dataset**

**Supplemental Data File 1.** Amplicon EZ sequencing data.
