# Supplementary material for: Suppressing mosquito populations with precision guided sterile males
Source: Nat Commun. 2021 Sep 10;12:5374. doi: 10.1038/s41467-021-25421-w (PMC8433431; doi:10.1038/s41467-021-25421-w)
Supplement: Supplementary file 13 — Reporting Summary [file 41467_2021_25421_MOESM13_ESM.pdf]

nature research

Corresponding author(s): Omar S. Akbari

Last updated by author(s): Jun 25, 2021

Reporting Summary

Nature Research wishes to improve the reproducibility of the work that we publish. This form provides structure for consistency and transparency in reporting. For further information on Nature Research policies, see our Editorial Policies and the Editorial Policy Checklist.

Statistics

For all statistical analyses, confirm that the following items are present in the figure legend, table legend, main text, or Methods section.

n/a	Confirmed	
☐	☒	The exact sample size (*n*) for each experimental group/condition, given as a discrete number and unit of measurement
☐	☒	A statement on whether measurements were taken from distinct samples or whether the same sample was measured repeatedly
☐	☒	The statistical test(s) used AND whether they are one- or two-sided<br>*Only common tests should be described solely by name; describe more complex techniques in the Methods section.*
☐	☒	A description of all covariates tested
☐	☒	A description of any assumptions or corrections, such as tests of normality and adjustment for multiple comparisons
☐	☒	A full description of the statistical parameters including central tendency (e.g. means) or other basic estimates (e.g. regression coefficient) AND variation (e.g. standard deviation) or associated estimates of uncertainty (e.g. confidence intervals)
☐	☒	For null hypothesis testing, the test statistic (e.g. *F*, *t*, *r*) with confidence intervals, effect sizes, degrees of freedom and *P* value noted<br>*Give P values as exact values whenever suitable.*
☒	☐	For Bayesian analysis, information on the choice of priors and Markov chain Monte Carlo settings
☐	☒	For hierarchical and complex designs, identification of the appropriate level for tests and full reporting of outcomes
☒	☐	Estimates of effect sizes (e.g. Cohen's *d*, Pearson's *r*), indicating how they were calculated

Our web collection on statistics for biologists contains articles on many of the points above.

Software and code

Policy information about availability of computer code

Data collection	LAM25 Locomotor Activity Monitor and DAMSystem3 software (TriKinetics), Noldus Ethovision XT 16
Data analysis	Prism 9.1 for MacOS (GraphPad Software), Noldus Ethovision XT 16

For manuscripts utilizing custom algorithms or software that are central to the research but not yet described in published literature, software must be made available to editors and reviewers. We strongly encourage code deposition in a community repository (e.g. GitHub). See the Nature Research guidelines for submitting code & software for further information.

Data

Policy information about availability of data

All manuscripts must include a data availability statement. This statement should provide the following information, where applicable:

- Accession codes, unique identifiers, or web links for publicly available datasets
- A list of figures that have associated raw data
- A description of any restrictions on data availability

Complete maps and plasmid DNA assembled in the study are deposited at Addgene.org (#164846 - #164848) and available for distribution (Table S18). All data used to generate figures are provided in the Supplementary Materials and Source Data files with this paper (Table S1-S18).

Field-specific reporting

Please select the one below that is the best fit for your research. If you are not sure, read the appropriate sections before making your selection.

☒ Life sciences ☐ Behavioural & social sciences ☐ Ecological, evolutionary & environmental sciences

For a reference copy of the document with all sections, see nature.com/documents/nr-reporting-summary-flat.pdf

Life sciences study design

All studies must disclose on these points even when the disclosure is negative.

Sample size	Sample size was chosen consistent with the previous literature reporting similar assays (e.g., Nature Biotech 2018,36:1062- 1066; eLife 2020,9:e51701). Sample size was maximised within the feasibility of performing biological assays with live insects. At least 50 individual males / females were independently scored / assessed in each replicate sample.
Data exclusions	No data were excluded from the analyses.
Replication	At least three biological replicates were performed as described in the main text, and no replicate was excluded.
Randomization	Organisms were randomized into their experimental groups.
Blinding	Blinding was not necessary in this study because every transgenic line and induced phenotype can be easily distinguished.

Reporting for specific materials, systems and methods

We require information from authors about some types of materials, experimental systems and methods used in many studies. Here, indicate whether each material, system or method listed is relevant to your study. If you are not sure if a list item applies to your research, read the appropriate section before selecting a response.

Materials & experimental systems

n/a	Involved in the study	
☒	☐	Antibodies
☒	☐	Eukaryotic cell lines
☒	☐	Palaeontology and archaeology
☐	☒	Animals and other organisms
☒	☐	Human research participants
☒	☐	Clinical data
☒	☐	Dual use research of concern

Methods

n/a	Involved in the study	
☒	☐	ChIP-seq
☒	☐	Flow cytometry
☒	☐	MRI-based neuroimaging

Animals and other organisms

Policy information about studies involving animals; ARRIVE guidelines recommended for reporting animal research

Laboratory animals	Aedes aegypti, Liverpool strain. all developmental stages and both sexes were analysed in the study.
Wild animals	no wild animals were used in the study.
Field-collected samples	no field collected samples were used in the study.
Ethics oversight	The study did not require an ethical approval

Note that full information on the approval of the study protocol must also be provided in the manuscript.
