# Supplementary material for: Suppressing mosquito populations with precision guided sterile males
Source: Nat Commun. 2021 Sep 10;12:5374. doi: 10.1038/s41467-021-25421-w (PMC8433431; doi:10.1038/s41467-021-25421-w)
Supplement: Supplementary file 11 — Editorial Assessment Report [file 41467_2021_25421_MOESM11_ESM.pdf]

nature portfolio

GUIDED OPEN ACCESS
nature genetics   nature communications   communications biology

Editorial Assessment Report

Dear Professor Akbari,

Thank you again for choosing to submit your manuscript using the Guided Open Access pilot at the Nature Portfolio. As part of this process, our editorial team has considered your paper for three of our journals with strong interest in publishing in your field: *Nature Genetics*, *Nature Communications*, and *Communications Biology*.

Your manuscript entitled "Eliminating Mosquitoes with Precision Guided Sterile Males" has now been reviewed by 4 experts in transgenic insect models, genetic engineering, and ecological modeling, whose comments are included in the attached Editorial Assessment Report. As part of the Guided Open Access pilot, editors from all 3 journals have discussed the reviewer reports and the manuscript's suitability for our journals. After careful evaluation, our editorial recommendation is to revise the manuscript and submit back through the Guided Open Access submission portal for consideration at *Communications Biology* or *Nature Communications*. Provided the revisions satisfy all technical and editorial concerns, *Communications Biology* is especially interested in publishing your manuscript. Please see details in the attached Editorial Assessment Report.

In brief, for publication in *Communications Biology*, we would require you to address the referee concerns regarding the fitness of pgSIT mosquitoes, scalability and advantages of this approach to other methods, and whether the modelling parameters are appropriate. For consideration in *Nature Communications*, the editors would require all reviewer concerns to be fully addressed. You will find more details regarding the required revisions in the Editorial Assessment Report below.

Please note that the Editorial Assessment Report is a standalone document that contains an editorial evaluation, recommendation and portable peer advice to help you navigate and interpret the reviewers' reports. It also provides guidance for adhering to best practice with regard to transparency and reproducibility, for example on the issue of sharing data. We have also included information about data accessibility and reproducibility, which we hope you find useful.

Should you have any questions about the recommended journals or would like advice on the revisions, you can contact me directly and I will be happy to assist. We look forward to receiving the revised version of your manuscript.

Yours sincerely,

George Inglis
--
George Inglis
Associate Editor
Communications Biology

On behalf of the Guided OA editorial team

Contents of this Report

- **Manuscript overview**: details about your manuscript and the editorial team.
- **Manuscript assessment**: personalised recommendation from the editors.
- **About the editorial process**: an overview of the Guided Open Access process.
- **Annotated reviewer comments**: the referee reports with comments from the editors.
- **Open research evaluation**: advice for adhering to best reproducibility practices.

Manuscript overview

Manuscript number	Submission date	Decision date
GUIDEDOA-21-00081	29 March 2021	5 May 2021

**Title**	Eliminating Mosquitoes with Precision Guided Sterile Males	**Corresponding author**	Omar Akbari<br>ORCID: ORCID
**Preprint information**	A preprint of this paper was posted on bioRxiv on March 6, 2021 https://www.biorxiv.org/content/10.1101/2021.03.05.434167v2	**Peer review type**	Single-blind
**Editorial Assessment Team**	**Primary editor: George Inglis**<br>**Home Journal:** *Communications Biology*, ORCID: 0000-0002-9069-5242<br>**Editorial team members:**<br>Ross Cloney, *Nature Communications*, ORCID: 0000-0003-3415-5468<br>Wei Li, *Nature Genetics*, ORCID: 0000-0002-7885-1775		
**About your primary editor**	George received his PhD in Genetics and Molecular Biology from Emory University, where he studied mouse models of voltage-gated sodium channel dysfunction and epilepsy. He also has research experience in epigenomics and in vitro models of neuronal development. George joined the editorial team of Communications Biology in September 2020 and is based in the New York office.		

Manuscript assessment and recommendation	
**Editor's summary of the manuscript and overall assessment**	Here, the authors have developed pgSIT, a CRISPR-based editing approach, in the mosquito, *Aedes aegypti*. Briefly, pgSIT utilizes a dominant CRISPR-based approach to generate sterile males that are deployable at any life stage, to suppress populations. The authors demonstrate that pgSIT does not substantially affect mosquito fitness, yet successfully disrupts genes essential for spermatogenesis and female flight. They use multigenerational cage trials and theoretical modeling in a wild population to demonstrate that pgSIT progeny can efficiently compete and eliminate mosquito populations, suggesting it could be applicable for real-life population control.<br><br>While the editors jointly decided to send this manuscript out to review based on the extensive modeling, we recognized that pgSIT was initially applied toward *Drosophila* in reference #9 (Kandul et al. 2019, *Nat. Comms*.). While we recognize the value of using pgSIT in a different system, the fact that pgSIT had previously been developed in *Drosophila* limited the novelty of the approach and prohibited further consideration by *Nature Genetics*.<br><br>The referees largely find the study to be well-designed and of interest, but there are some concerns regarding how the theoretical modelling was established (Referees #1-2) and the relevance of this approach to real-world environments (Referees #2-4). In particular, there are several questions about whether the technique would be scalable and suitable for urban areas, especially given that not all pgSIT mosquitoes are infertile. Referee #2 also comments on whether pgSIT mosquito fitness is a robust phenotype. Altogether, pgSIT appears to be a promising advance for mosquito control and may spark further discussion, though it is unclear whether it could feasibly be implemented in the field.<br><br>In summary, a resubmission should include the following revisions:<br>(1) Justify the parameters used for the theoretical population control simulation, and overall compare pgSIT to previous approaches, as highlighted by Referees #1-3. Please also clarify whether MGDrivE is a suitable algorithm for pgSIT mosquitoes.<br>(2) As noted by Referees #2-4, qualify statements regarding the fitness of pgSIT mosquitoes, advantages of this approach, and likelihood of real-world applicability. We also believe it would be important to discuss the scalability of this approach.<br>(3) Please elaborate on the context and discussion of background literature, as outlined by Referee #2.

Editorial assessment overview

Nature Genetics	Not feasible to meet the editorial requirements	The technical and conceptual advance (compared to prior literature) represented by the method is not sufficient for publication in *Nature Genetics*.
Nature Communications	Some additional data needed, but generally minor revisions	The adaptation of existing methodology into a relevant species is interesting, but further consideration would require all reviewer concerns to be fully addressed.
Communications Biology	Minor revisions needed	As noted by the reviewers, improved discussion of mosquito fitness, pgSIT scalability, and the modeling approach, would be required for publication.

**Editor's recommendation**	***Option 1*: Revise for consideration at Communications Biology**<br>While many revisions are needed, these do not require any new datasets of expansion of the work beyond proper justification of methods and discussing the limitations of the approach.<br><br>***Option 2*: Revise and submit to Nature Communications**<br>For publication in *Nature Communications*, the authors would need to fully address all reviewer concerns, including the advantages over similar methods and the broader applicability of the approach.<br><br>**Please state in your cover letter which journal you have revised for.**
**Next steps**	If you would like to follow our recommendation, when you are ready you can upload the revised manuscript, along with your point-by-point response to the reviewer's reports and editorial advice here*.

About the editorial process

By selecting the Nature Portfolio Guided Open Access option, your manuscript was assessed for suitability in three of our titles that provide venues for publication of high-quality work across the spectrum of genetics research: *Nature Genetics, Nature Communications*, and *Communications Biology*. For more information about Guided Open Access, please see here.

Collaborative editorial assessment

Your editorial team discussed the manuscript to determine its suitability for the Nature Portfolio Guided OA pilot. Our assessment of your manuscript takes into account several factors, including whether the work meets the **technical standard** of the Nature Portfolio and whether the findings are of **immediate significance** to the readership of at least one of the participating journals in the Nature Portfolio Guided Open Access genetics cluster.

Peer review

Experts were asked to evaluate the following aspects of your manuscript:

Novelty in comparison to prior publications;

Likely audience of researchers in terms of broad fields of study and size;

Potential impact of the study on the immediate or wider research field;

Evidence for the claims and whether additional experiments or analyses could feasibly strengthen the evidence;

Methodological detail and whether the manuscript is reproducible as written;

Appropriateness of the literature review.

Editorial evaluation of reviews

Your editorial team discussed the potential suitability of your manuscript for each of the participating journals. They then discussed the revisions necessary in order for the work to be published, keeping each journal's specific editorial criteria in mind.

Journals in the Nature portfolio will support authors wishing to transfer their reviews and (where reviewers agree) the reviewers' identities to journals outside of Springer Nature. For any questions about review portability, please contact our editorial office: guidedoa@nature.com

Annotated Reviewer Reports

The editor has included some additional comments on the specific points raised by the reviewers below. However, please note that all points should be addressed in a revision, even if the editor has not specifically commented on them.

Reviewer #1

Report received: 20 April 2021

**Reviewer #1**	Michael Smanski, Ph.D.<br>University of Minnesota
**Reviewer #1 expertise**<br>*Summarised by the editor*	This reviewer has expertise in insect genome engineering.
**Editor's comments about this review**	While this Reviewer provided positive feedback about the novelty and quality of the study, the editors at *Nature Genetics* felt the conceptual advance in translating pgSIT from *Drosophila* to *Aedes aegypti* was insufficient for further consideration. Primarily this Reviewer had concerns about the rationale behind the theoretical modeling approach, and offers several helpful suggestions to improve the clarity of the Methods and Discussion.
**Reviewer #1 comments**	
**Overview**	The authors present data describing the performance of their pgSIT approach to population suppression in the applied organism, *Ae. aegypti*. pgSIT provides a mechanism to generate sterile males without the use of irradiation. Two parental lines (one homozygous for Cas9 and one homozygous for sgRNAs targeting female flight and male fertility) are crossed to generate sterile males, that can be released to compete with wild-type males to mate with wild-type females. In this manuscript, the authors describe a complete story, from design and testing of genetic components to validate gene targets, construction of pgSIT mosquitoes to test their performance and fitness, extensive cage-based population suppression experiments, and simulation modeling for a real-world release scenario.<br><br>The data is of very high quality and supports the conclusions drawn. There are extensive and detailed results presented in the supplementary figures that allow interested readers to 'dig in', but the overall story presented is clear and easy to follow. The figures are well-organized with a few minor suggestions noted in my critiques below. The only major revision requested is to provide more details and justification for the parameters used during simulation modeling.

	This paper is of very high impact. While a proof of concept was previously published in flies, the demonstration of the technology in an applied insect species is of substantial impact, and is by no means trivial. My understanding of the Guided OA request to review is that the editors have already made a decision to pass on *Nature Genetics* and are only considering this for *Nature Communications* or *Communications Biology*. I would request that the editors reconsider and instead publish this in *Nature Biotechnology* or *Nature Genetics*, which are a better fit because of the quality, impact, and widespread interest in this work.<br><br>There are suggested improvements listed beneath my signature. I recommend this paper for publication in *Nature Communications* with minor revisions. I welcome questions or concerns about the content of this review by the authors of the manuscript or editors. If it is against editorial policy to have non-anonymous reviews, please remove my name below.<br><br>Best,<br>Mike Smanski<br>smanski@umn.edu

Specific comments

#	Reviewer comment	Editorial comment
**Major Concerns:** There are several details regarding the modeling that should be revised, better justified, or better explained.		
**1**	First, please justify the use of 16 mosquitoes per human structure (and no mosquitoes outside of human structures) for the simulation. The total number of adult mosquitoes at equilibrium in this model is less than 1000. A previous modeling paper using the same site (Strugarek et Dumont 2018) estimate the population of adult males on the islet to be greater than 5000 (so the total adult population would be ~10,000, or 10x higher than used in this study).	In line with our commitment to reproducible science, a revision for *Nature Communications* or *Communications Biology* should fully justify the experimental design and model parameters (as further described in points #2-3 below). Reviewer #2 also shares several comments regarding the modeling approach.
**2**	Second, it is unclear if the 400 pgSIT eggs released per adult mosquito are only male or are male plus female eggs. Please be more explicit. If it is male plus female, then presumably the pgSIT females will survive to adulthood and the subpanels in Figure 4B need to be updated to clarify that the y-axis is no. of wild-type adult female *Ae. aegypti*.	
**3**	Lastly, more details on egg hatch rate in the model should	

	be included in the main text. The authors state that releasing 400:1 pgSIT:wild-type is similar to a 10:1 release ratio of adults, but this similarity depends a lot on the modeled survival rate of their released eggs (which presumably would be hatched from containers with greater survivability than wild laid eggs). If the 400 released eggs are all male, it would be most appropriate to describe this as a 800:1 release of pgSIT males to adult males.	
**Minor Concerns:**		
**4**	**Line 77:** Change *Aegypti* to *aegypti*.	
**5**	**Line 104:** Would be interesting to mention that the maternal Cas9 was better in all of the measurable comparisons (i.e those where both versions worked 100%).	
**6**	**Line 598:** Elsewhere (e.g. figures) ratios are given as pgSIT:Wild-type, while here the inverse is given. Better to keep it consistent and list these as 1:1, 5:1, 10:1, etc.	
**7**	**Figure 1B and E:** I get why you are showing both the maternal and paternal cas9/sgRNA on the right side of these panels, but it is still somewhat confusing why you are showing both types of crosses. You don't show the maternal and paternal for the other controls, and numbers for the maternal vs paternal for the two cas9+sgRNA are not distinct. It seems more straight forward to just show the compiled results and say "there was no difference when the Cas9 was paternal and the sgRNA maternal and vice versa".	
**8**	**Figure 1B and E:** I think it would be better if you have a label to the left of the chromosome schematics, and label which chromosomes are shown (it seems like you drop Chr. 2, but this is not clear).	Explicitly adding chromosome labels would improve readability.
**9**	**Figure 1B and E (Legend):** You refer to B and E as histograms, but these are better described as bar graphs with each datapoint shown. (conventional histograms show a probability distribution across the x-axis variable broken into bins).	From an editorial standpoint, these images are bar graphs, rather than histograms (also see Reviewer #1, point #12).
**10**	**Figure 2B:** the y-axis of progeny survival (%) does not really work since the grey bars are not showing survival.	

	Better to just label the y-axis 'Percent', and keep the inset labels as they are.	
**11**	**Figure 2C:** I think you mislabeled the pgSIT mosquitoes being taken out. It should say 250 instead of 50.	
**12**	**Figure 2 (Legend):** Same comment about histograms from **Point 9**.	
**13**	**Figure 3A:** The eye colors in gRNA and pgSIT mosquitoes in the key are hard to see.	It may be useful to change the entire body color of gRNA or pgSIT mosquitoes in this diagram (rather than just the eyes).
**14**	**Figure 4:** The font and style for the subpanel labels A, B, C are different from the rest of the figures.	We would recommend changing the labels in all figures to lowercase letters (a, b, c), to match journal formatting styles.

Reviewer #2

Report received: 11 April 2021

**Reviewer #2**	This reviewer has not chosen to waive anonymity. The reviewer's identity can only be shared with representatives of an established journal editorial office.
**Reviewer #2 expertise** *Summarised by the editor*	This reviewer has expertise in the use of experimental and theoretical mosquito models, including. Population control techniques.
**Editor's comments about this review**	While we recognize that this Reviewer's language is candid at times, their feedback emphasized the need for authors to carefully qualify pgSIT mosquito fitness, scalability, and relevance to other approaches. Taken together with comments from Reviewer #1, we believe this review highlights the need to better contextualize the pgSIT simulation and overall fitness of progeny.
**Reviewer #2 comments**	
**Overview**	The paper brings a very interesting approach on how to obtain completely sterile males and removal of 100% females.<br><br>The manuscript attempts to bring a fulfillness and grandiosity intimidating the readers and leading them to a mental confusion and accepting everything that was presented.

Specific comments

#	Reviewer comment	Editorial comment

**1**	The title is misleading for something not reflected in the manuscript results. Half of the results are missing in the abstract section, with no mention about the flightless mosquitoes nor the combination of the two strategies.	We would recommend changing the title to "Controlling mosquito populations", or something less definitive than "eliminating"<br><br>Currently, the abstract is at 124 words. Given that we typically suggest a limit of ~150 words for this section, there is room to expand on the results of study.
**2**	For the figures, all legends are incomplete. And the figures are extremely hard to comprehend and pollute.	We believe the Reviewer means that all figure legends should explicitly state sample sizes, and that some figures are very dense. We would recommend splitting up Figures to improve readability. For instance, Fig. 1a-b and Fig. 1c-e could be separated into their own figures (Fig. 1-2).
**3**	For the supplementary material should complement the manuscript and bring useful information, but the way is presented is that every single possible way to present something that sound as a result was included. And their majority could have been included in the text. This reflects a confusing thinking about the supplementary material selection. The main figures are so crowded that part of them should be the supplementary material, replacing most of the current ones.	Up to 10 figures are allowed in the main text, so there is room for more data to be included from the Supplementary Information. However, we would leave this point to the authors' discretion.
**4**	**Lines 117-120:** What is the potential reason for the other non-selected strains to have variable fertility levels and even full fertility? Same for flying females of non-selected pgSIT strain.	
**5**	**Lines 125-128:** Why authors were so confident that they would have superior results and "robust" transgene expression? Specially, when other strains obtained with the same construction are fertile (Figure S2) and some females even though they were flightless, some were still able to blood feed (Figure 3).	Reviewer #3 also noted that a subset of mosquitoes are still fertile. Please address this limitation in the text and qualify statements regarding the "robustness" of this method.
**6**	**Lines 132-134:** Linked to the previous comment and the speculative sentence on these lines, the potential application of egg release for population suppression, and given the unrealistic possibility that these females may	Reviewers #3-4 also comment on the need for a broader discussion on the limitations of this approach and barriers to real-life application.

	find conditions to mate, blood feed and lay eggs, how would be the potential impact of these offspring? Since regulatory bodies may find this information quite alarming.	
7	**Lines 137-142:** It's known that is not only time but also the moment that the coitus is interrupted so a second male can contribute with sperm to the offspring (DOI: 10.1371/journal.pone.0193164). By number used at the beginning of the experiment (which is also not clear the methods) the male sexual harassment over females may be very high, and within the 2-6 hours, males keep removing each other during coitus or several males trying to mate at same time with a flying female, causing the tendency of females not to move/fly to avoid coitus. The other possibility is the mating vigor (which is not the mating capacity as shown) and males need longer periods to mate.	
8	**Lines 151-154:** If as stated in the manuscript, no significant impact on the fitness cost was found, however comparing these result with a study using ionizing gamma radiation on male mosquitoes, in both studies the population suppression took 6 generations to collapse using the same sterile : fertile ratio (DOI: 10.1098/rstb.2019.0808), and using eggs for releases indicates a higher impact on fitness by the use of much higher ratios (20:1 or 40:1) for the same number of generations to reach population suppression.	Please qualify the overall fitness of pgSIT mosquitoes and compare to the reference provided here.
9	**Lines 164-167:** As there were no field releases, why 25% reduction in male competitiveness was chosen? Another indication that the pgSIT males have lower fitness, is the comparison with a real field release in Brazil performing a mark-release-recapture experiment using drones and gamma irradiated males, in which within couple of releases (in the same period) they manage to observe more than 50% reduction of viable eggs in the field compared with the control site (DOI: 10.1126/scirobotics.aba6251).	
10	**Line 181:** I am skeptical to call the strain 100% fit.	Given the concerns about fitness from Reviewer #2, these claims should be qualified.
11	**Lines 181-183:** The cage suppression experiment is based on a discrete and multigenerational process, which is	We believe this point should be mentioned as a limitation of the

	quite artificial and not realistic. A similar experiment established a wild population in the semi-field cages, before starting releasing their "sterile" males. And because they also followed control cages during the study period, the replicates showed it is clear the impact and the low variability among the same sample group (DOI: 10.1038/s41586-019-1407-9).	method.
**12**	**Lines 183-186:** Excessive negative fitness cost can be a problem during operational releases, even in a small trial. The transgenic strain used in Brazil (OX513A) was known to have negative fitness cost (DOI: 10.1371/journal.pone.0020699 and DOI: 10.1371/journal.pone.0026086). And the outcome of this was seen overlapping the studies using this same strain for mass production and population suppression (DOI: 10.3791/3579 and DOI: 10.1371/journal.pntd.0003864). Although the authors claim to have achieved population suppression around 70 to more than 90%, the released area was quite small (5.5 ha with around 500 residences) and required a production of 0.5 million males/week to reach this result.	
**13**	**Line 188:** It is written "realized" but do you mean "released"?	
**14**	**Line 192:** females produce 450 eggs throughout their life, this is a very high assumption since this would be around 90 eggs/ gonotrophic cycle, which may not be realistic depending on strain, blood quality, oviposition site and other factors.	
**15**	**Lines 198-199:** Although field models show promising results using pgSIT, the scalability was not modeled and tested under realistic mass rearing facility to attend areas of pilot trials that are slightly more challenged than Onetahi island.	Reviewers #3-4 also shared concerns about scalability that should be discussed.
**16**	**Line 200:** Senecio's technology so far is not available for operational trials, and Debug haven't tried transferring their technology to other operational trials with bigger populations and the maximum mentioned was less than 3 million males/week, which depending on the trial site is not enough to suppress the population.	
**17**	**Lines 204-207:** Egg releases sound not as a promising technique since there will be no control on the male quality produced. And the dissemination of these eggs	

	might be as laborious as adult production and release, unless the target is only to produce a product and not vector control. How would be the dynamics of deploying dissemination stations and how would be the entomological surveillance of these areas?	
**18**	**Line 201-214:** However, the proposed model also showed that egg release was less effective as adult releases, meaning that the suppression delay will persist.<br>Figure 4: According to some quick calculations using data from the manuscript only for Onetahi, which seems to be a quite isolated area and without a real mosquito burden and disease transmission, using the pgSIT it would be necessary to obtain 0.8 million eggs of the strain to be released every week. And a facility to keep this production that depends on two strains to obtain, will definitely be a complex process since they must be independent facilities, multiplying by two the area, team, equipment all consumables in order to produce for a quite small trial.	

**Reviewer #3**<br>*Report received:* 28 April 2021	
**Reviewer #3**	This reviewer has not chosen to waive anonymity. The reviewer's identity can only be shared with representatives of an established journal editorial office.
**Reviewer #3 expertise**<br>*Summarised by the editor*	This reviewer has expertise in transgenic *Drosophila* models.
**Editor's comments about this review**	This Reviewer reaffirmed that pgSIT could be conceptually interesting to the field, but provided insightful feedback on the scalability of this system and whether it would be applicable to real-world situations. Altogether, the comments from this Reviewer emphasize the need to elaborate on limitations and scalability of pgSIT.
**Reviewer #3 comments**	
**Overview**	This manuscript describes a new implementation of Cas9 to generate *Aedes* mosquitoes that, it is hoped, can be released into wild populations as embryos, leading to elimination of those populations. Given the moniker "precision-guided sterility," the rapid pace of theoretical and experimental improvements in Cas9 gene drive systems, and the senior author's expertise in this realm, I expected this would

	describe a multi-component, controlled gene drive system. In actuality, it is a straightforward but well-designed use of Cas9 to generate mutations in two target genes, of which is required for male fertility and the other for female wing posture and therefore flight. Transgene lines that, when crossed to a Cas9-expressing line, gave nearly complete male sterility and female flightlessness were selected and analyzed. The use of the female flightlessness is an important addition that means mosquitoes will not need to be sex-sorted after production.<br><br>The approach outlined here has some distinct advantages over release of sterile males in the RIDL approach and release of Wolbachia-infected males. It has the disadvantage of involving transgenic mosquitoes. It is unclear whether it will ultimately be successful in getting approval, but it is important that the method be published so it can be part of the discussion. I expect that publication of this work will lead to substantial discussion.<br><br>The work is generally well documented and controlled, with appropriate rigor. There are a few issues that require additional discussion:

Specific comments

#	Reviewer comment	Editorial comment
**Major Concerns:**		
**1**	An important question that is not discussed fully concerns persistence of pgSIT in the environment. Fig 1B shows that there are rare males that are fertile. Scaling up to the numbers proposed for release suggests that the probability that the transgene will introduced into the local population is not zero. Of course, if the population is completely eliminated after several rounds of releases, then rare fertile males or flight-capable females are not a problem. However, this possibility (which is not a problem for RIDL, as it does not involve transgenic animals) likely does raise the bar on what probability of population elimination will be considered acceptable to obtain approvals.	
**2**	I am not an expert in modeling, but MGDrivE appears to have been developed for gene drive. Was this used previously to simulate successive releases, such as of Wolbachia-infected males?	Please clarify whether MGDrivE is also compatible with the pgSIT model and justify its usage.
**Minor Concerns:**		

**3**	I was initially put off by the use of male and female symbols in text, but I will admit that it does make it easier to read. However, the use of ♂'s (I used the male symbol here, in case it does not reproduce in final comments) is a possessive, not a plural. The standard genetic nomenclature for plural is to use two copies of the symbol adjacent to one another, as in ♂♂ (two consecutive male symbols; this may need to be defined at first use). I really don't think the symbols work as superscripts.	
**4**	**Line 80:** *"…we demonstrate resulting progeny of flightless ♀'s and fit sterile ♂'s that can compete…"* is confusing, as it sound like it is the progeny of flightless x sterile mosquitoes that can compete, rather than the progeny of the cross of the two strains, and that it is the sterile males can compete for mating.	
**5**	**Line 103:** It is confusing to report 0-94% fertility and then say two lines reached 100% sterility. (Actually, sterility and fertility are not qualitative traits – an individual is one or the other. The quantitative trait is fecundity.)	
**6**	**Line 105:** 3 of 5 became a fraction	
**7**	**Line 116:** I don't think this is true introgression, just backcrossing. Also, "trans-homozygous" doesn't make sense as a word; these are double homozygotes (also in Fig. 2A).	
**8**	**Line 120:** This doesn't require a response unless it is an error, but it seems backwards to determine copy number and transgene location after building the double homozygote rather than before.	
**9**	**Fig. S1B:** Proteins were compared but the legend uses italics and even says "betaTub gene."	Per journal style, only gene names should be written in *italics*.
**10**	**Fig. S7:** Panels need to be labeled.	Please label each panel with a letter (e.g. Fig. S7a-c).
**11**	**Fig. S8 and S9:** Panel B doesn't seem to be helpful. Is this just showing low coverage of ONP reads across this exon? There is no description of Oxford Nanopore sequencing of mutations provided (there is only ONP of transgene insertion sites and copy number). "Target sites" is really gRNA targeting sequence. A line showing the actual cut site (at least on the strand shown) would help.	All protocols and analyses should be fully described in the Methods.
**12**	**Reproducibility:** Statistical tests are appropriate and	

	sufficient detail is given in Methods to allow reproduction (with the exception of the Oxford Nanopore sequencing mutations being missing).	

Reviewer #4
Report received: 22 April 2021

**Reviewer #4**	This reviewer has not chosen to waive anonymity. The reviewer's identity can only be shared with representatives of an established journal editorial office.
**Reviewer #4 expertise** *Summarised by the editor*	This reviewer has expertise in genetic mosquito models.
**Editor's comments about this review**	Similar to Reviewer #3, this Reviewer also provides useful feedback highlighting the need for better context for the modelling approach, and further discussion of whether it could be scaled for real-world applications. Taken together, these reviews emphasize the need for an extended Discussion of method limitations and broader applicability.

Reviewer #4 comments

**Overview**	The Akbari laboratory has previously reported on an elegant CRISPR/Cas9 approach for population suppression of the fruit fly *Drosophila melanogaster*. This manuscript reports on the adaptation of this approach to the *Ae. aegypti* mosquito.<br><br>The work is technically sound, and adequately detailed. Comments follow.

Specific comments

#	Reviewer comment	Editorial comment
1	In **Figure 2C**, mating was allowed to occur for different lengths of time. However, missing is the time of the day at which each experiment was initiated, as mating is not uniform in any 24-hour period, as it is controlled by the circadian clock. The time of the day at which each experiment was initiated needs to be specified.	Please confirm the time of day and address whether circadian rhythm could be a confound.
2	**Figure S4** is difficult to understand. There are boxes with arrows but some of the fluorescence is not visible. Also,	

	the transmitted light images are often poor and it is not clear which organ is the source of fluorescence.	
**3**	Are the data in **Fig. S5F** the same as in **Fig. 2B**? If not, what is the difference?	
**4**	**Important:** Whereas the modelling of **Fig. 4** seems to be OK (but I am not an expert), it needs to be put into context in the Discussion. The island is very small, about 2 sq. miles. It is not clear if this figure is for the Teti'aroa atoll, or whether the Onetahi islet is even smaller. Please state the actual figure in the Discussion. The population is also very small (about 100 persons, is this correct??), and this should also be specified in the Discussion.	
**5**	To put the findings into context, the Discussion should comment on:<br>i. The logistics to implement this strategy in a city where major dengue or Zika epidemies occur.<br>ii. Given that this is a population reduction approach (as opposed to population elimination), the need of implementation of this strategy on a continuous basis (and possible costs) should be considered.	These are critical points for Discussion and nicely summarize concerns from Reviewers #2-3.
**6**	In summary, this manuscript adapts from *Drosophila*, an elegant strategy for suppression of *Aedes* populations. The experiments are expertly executed, and the modelling is probably sound. However, considerations of what would it take to implement this strategy in a major endemic area should be clearly considered in the Discussion.	
**7**	This work offers an alternative strategy for mosquito population reduction, that has several advantages mentioned by the authors.<br><br>However, as pointed out, I feel it crucial that the possible shortcomings also need to be discussed, to provide a balanced view that the readers can evaluate.	

Open Research Evaluation	
**Data Availability**	
**Data Availability Statement**	Our journals strongly support public availability of data and custom code associated with the paper in a persistent repository where they can be freely and enduringly accessed or as a supplementary data file when no appropriate repository is available. If data and code can only be shared on request, please explain why in your data Availability Statement, and also in the correspondence with your editor. For more information, please refer to https://www.nature.com/nature-research/editorial-policies/reporting-standards#availability-of-data<br><br>Please ensure that datasets deposited in public repositories are now publicly accessible, and that accession codes or DOI are provided in the "Data Availability" section. As long as these datasets are not public, we cannot proceed with the acceptance of your paper. For data that have been obtained from publicly available sources, please provide a URL and the specific data product name in the data availability statement. Data with a DOI should be further cited in the methods reference section.<br><br>See our policy page for details.
**Mandatory data deposition**	Mandatory deposition is required for DNA and RNA sequences and sequencing data. For an up-to-date list of approved repositories for each mandatory data type, please visit https://www.springernature.com/gp/authors/research-data-policy/repositories/12327124. In particular, we recommend the Gene Expression Omnibus.<br><br>For more information on mandatory data deposition policies at the Nature Portfolio, please visit http://www.nature.com/authors/policies/availability.html#data<br><br>Accession code(s) for deposited data must be provided in the Data Availability statement in the final version of the paper. Failure to do so will delay publication. Please ensure data are

	available prior to publication.<br>http://www.nature.com/authors/policies/availability.html#data
**Source data**	The following figure panels should be accompanied by the underlying source data: **Fig. 1b/e, Fig. 2b-e, Fig. 3c, Supp. Fig. S2c, Fig. S3c, Fig. S5, Fig. S7, Fig. S10b-e**<br>Source data files will be mandatory prior to publication in a Nature Portfolio journal.
**Data citation**	Where datasets are hosted in public repositories that provide datasets with Digital Object Identifiers (DOIs), we encourage these datasets to be formally cited in reference lists. Citations of datasets, when they appear in the reference list, should include the minimum information recommended by DataCite and follow journal style. For example:<br>Hao, Z., AghaKouchak, A., Nakhjiri, N., Farahmand, A. Global Integrated Drought Monitoring and Prediction System (GIDMaPS) Data sets. *figshare*. http://dx.doi.org/10.6084/m9.figshare.853801 (2014)<br>Citing and referencing data in publications supports reproducible research, by increasing the transparency and provenance tracking of data generated or analysed during research. Citing data formally in reference lists also helps facilitate the tracking of data reuse and may help assign credit for individuals' contributions to research. A number of Springer Nature imprints are signatories of the Joint Declaration on Data Citation Principles, which stress the importance of data resources in scientific communication.
**Materials availability**	To improve transparency, we suggest adding a statement about the availability of pgSIT mosquito strains. See our policy page for details.
**Code Availability**	
**Code Availability**	See our policy page for details.

**Code Citation**	In addition to making the custom code available, we ask that you ensure that the version of the code/software described in the paper is deposited in a DOI-minting repository (eg, Zenodo) and that this DOI is also cited in the main Reference list. See here for details.
**Display Items**	
**Data Presentation**	Please ensure that data presented in a plot, chart or other visual representation format shows data distribution clearly (e.g. dot plots, box-and-whisker plots). When using bar charts, please overlay the corresponding data points (as dot plots) whenever possible and always for n ≤ 10. (Please see the following editorial for the rationale behind this request and an example https://www.nature.com/articles/s41551-017-0079).
**Figure Legends**	**Figures 1b, 1e, 2b, 2c-2e (bottom panels), Supplementary Figures 5a-5f, 7 (right panel):** Wherever statistics have been derived (e.g. error bars, box plots, statistical significance) the legend needs to provide and define the n number (i.e. the sample size used to derive statistics) as a precise value (not a range), using the wording "n=X biologically independent samples/animals/cells/independent experiments/n= X cells examined over Y independent experiments" etc. as applicable. Please update the figure legends accordingly.<br><br>**Figures 1b, 1e, 2b, 2c-2e (bottom panels), Supplementary Figures 4a-4c, 4e-4f:** Please note that the exact p value should be provided, when possible.<br><br>**Figure 1c, Supplementary Figures 4a-4f:** Please state in the legends how many times each experiment was repeated independently with similar results. This is needed for all experiments, but is particularly important wherever results from representative experiments (such as micrographs) are shown. If space in the legends is limiting, this information can be included in a section titled "Statistics and Reproducibility" in the methods section.<br><br>**Figure 7 (right panel):** Please note that the box plots need to be defined in terms of minima, maxima, centre, bounds of

	box and whiskers and percentile in the legend.<br><br>**Supplementary Figures S6, S8-S9:** Please state the genomic coordinates and annotation in the figure legend.
**Micrographs**	Please ensure that all micrographs/panels include a scale bar and that this scale bar is defined on each the panels or in the figure legends. **Please update Figure 1c and Supplementary Figure 4a-f accordingly.**
**Research ethics**	
**Competing Interests**	In the interests of transparency and to help readers form their own judgements of potential bias, Nature Portfolio journals require authors to declare any competing financial and/or non-financial interests in relation to the work described.<br><br>Please provide a 'Competing interests' statement using one of the following standard sentences:<br>• The authors declare the following competing interests: [specify competing interests]<br>• The authors declare no competing interests.<br><br>**Please change the title of the "Disclosures" section to "Competing Interests"**<br><br>See our competing interests policy for further information: https://www.nature.com/nature-research/editorial-policies/competing-interests
**Methods assessment and reproducibility**	
**Statistical Evaluation**	• Please note that statistics such as error bars significance and p values cannot be derived from n<3 and must be removed in all such cases.<br>• We strongly discourage deriving statistics from technical replicates, unless there is a clear scientific justification for why providing this information is important. Conflating technical and biological variability, e.g., by pooling technically replicates samples across independent experiments is strongly discouraged. (For examples of expected description of statistics in figure legends, please see the following

	https://www.nature.com/articles/s41467-019-11636-5 or https://www.nature.com/articles/s41467-019-11510-4).<br>• All error bars need to be defined in the legends (e.g. SD, SEM) together with a measure of centre (e.g. mean, median). For example, the legends should state something along the lines of "Data are presented as mean values +/- SEM" as appropriate.<br>• All box plots need to be defined in the legends in terms of minima, maxima, centre, bounds of box and whiskers and percentile.<br>• The figure legends must indicate the statistical test used. Where appropriate, please indicate in the figure legends whether the statistical tests were one-sided or two-sided and whether adjustments were made for multiple comparisons.<br>• For null hypothesis testing, please indicate the test statistic (e.g. F, t, r) with confidence intervals, effect sizes, degrees of freedom and P values noted.<br>• Please provide the test results (e.g. P values) as exact values whenever possible and with confidence intervals noted.
**Digital image integrity**	Please pay close attention to our Digital Image Integrity Guidelines. Also ensure that you retain unprocessed data and metadata files after publication, ideally archiving data in perpetuity, as these may be requested during the peer review and production process or after publication if any issues arise.
**Methods Descriptions**	The Methods must contain sufficient detail such that the work could be repeated. It is preferable that all key methods be included in the main manuscript, rather than in the Supplementary Information.<br>Nature Portfolio journals encourage authors to share their step-by-step experimental protocols on a protocol sharing platform of their choice. Where such protocols are available, please provide a DOI or other citation details in the paper. The Nature Portfolio's *Protocol Exchange* is a free-to-use and open resource for protocols; protocols deposited in *Protocol Exchange* are citable and can be linked from the published article. More details can found at www.nature.com/protocolexchange/about.

**Study design and reporting**	We have included as an attachment to the decision letter a version of your Reporting Summary with a few notes. This is mainly for your information, but we hope it is helpful when preparing your revised manuscript. If you decide to resubmit the manuscript for further consideration, please be sure to include an updated Reporting Summary.
